# Spectacles with non‑refractive opaque features for paediatric myopia: a safety pilot trial

**DOI:** 10.1038/s41433-026-04795-5

**Published:** 2026-08-14

**Authors:** Ravi C. Bakaraju, Jennie Diec, Daniel Tilia, Klaus Ehrmann, Ranjay Chakraborty

**Affiliations:** 1 nthalmic pty ltd, Sydney, Australia; 2 School of Optometry and Vision Science, UNSW, Kensington, NSW Australia; 3 Myopia and Visual Development Lab, Caring Futures Institute, College of Nursing and Health Sciences, Flinders University, Adelaide, SA Australia

**Keywords:** Refractive errors, Paediatrics

## Introduction

Optical myopia-control strategies predominantly introduce competing defocus, extend depth of focus, or modify retinal contrast [1]. A single-vision spectacle lens utilising non-refractive, light-absorbing opaque features to alter spatiotemporal retinal luminance during natural eye movement without defocus, blur, or scatter was developed [2]. Visual performance in adults was superior to a competing defocus design [3]. In-vivo myopia efficacy is unknown, and experimental evidence that partial occlusion could potentiate myopiagenesis [4], underpinning the need for paediatric safety data. A contact-lens counterpart demonstrated safety in a small pilot [5]; however, spectacle delivery is preferable for younger children. This 14-month, three-phase, within-participant crossover pilot assesses safety (primary) and axial elongation (secondary) in myopic children.

## Methods

The study was conducted at five Australian sites with ethics approval and trial registration. Myopic children aged 7–15 years (<-0.50D) were enrolled; exclusion criteria included ocular pathology, binocular vision anomalies, and prior myopia-control treatment (Supplementary Figure 1). Test spectacles incorporated optical films bearing non-refractive opaque features ( ~ 950 features, 0.03 ×1.5 mm, across ~50 mm diameter) adhered to single-vision lenses matched in vertex power; control spectacles were identical without films. Phase 1 (0-6 months): control; Phase 2 (6–12 months): A.R.R.E.S.T. films; Phase 3 (12–14 months): control. Visits were 2–monthly.

The primary variable was non-cycloplegic axial length by non-contact optical biometry; cycloplegic data were not collected. Safety assessments included slit-lamp examination as required, high-contrast visual acuity (HCVA), and participant-reported comfort. Analysis was intention-to-treat (ITT). Linear mixed models with random intercepts for participant and eye nested within participant, adjusting for baseline age and axial length. Two participants (P04, P11) discontinued before Phase 2 completion; missing data were handled under a missing-at-random assumption. Phase was the fixed factor; growth slopes and matched 2-month increments were secondary outcomes.

## Results

**Fig. 1:**
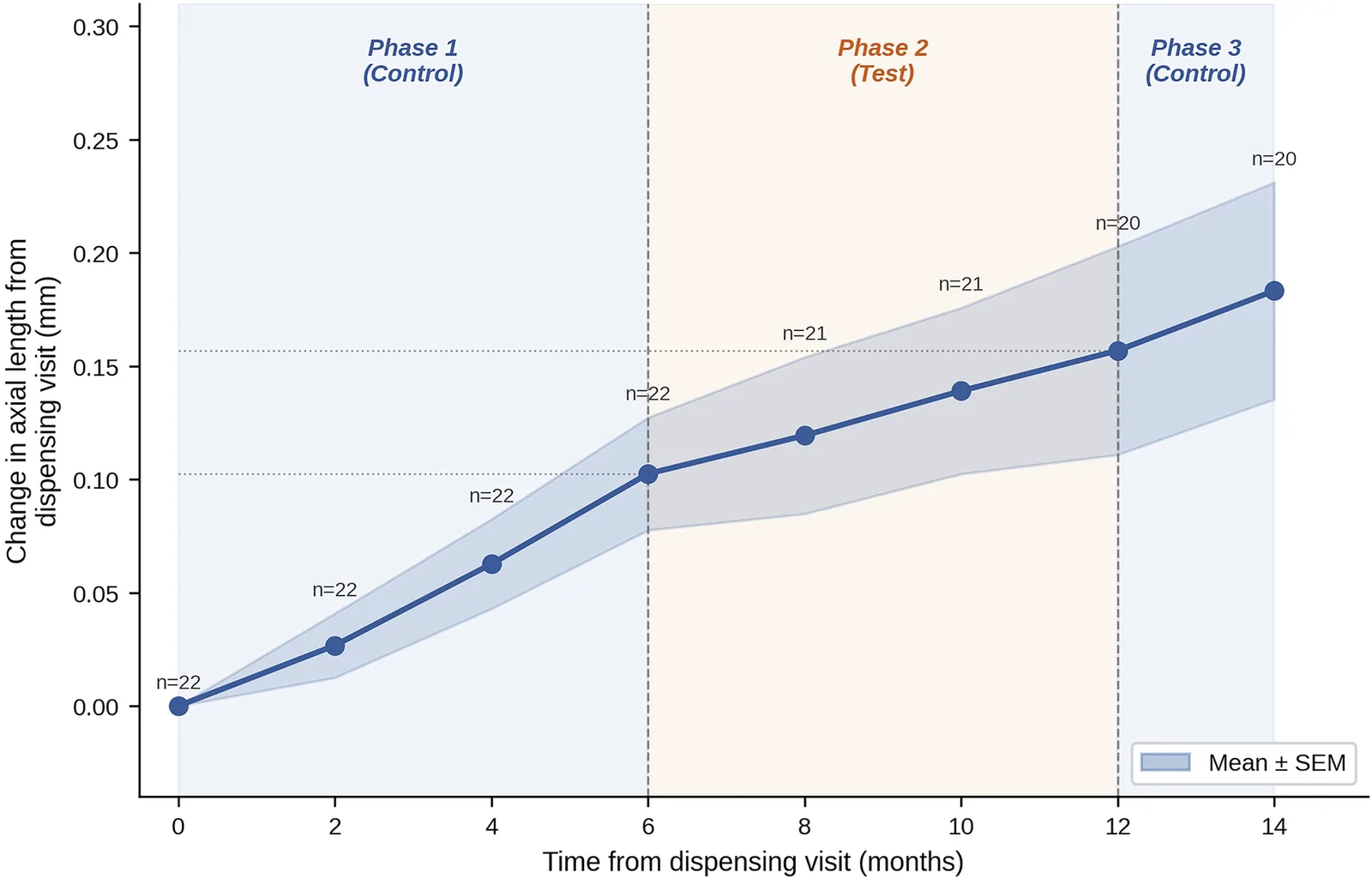
Group-level mean axial-length change (ΔAL, mm) from the Phase 1 dispensing visit across the 14-month study period (ITT; n = 22, both eyes combined). Data points represent participant-level means ± SEM at each 2-month visit. Phase 1 (0–6 months; bilateral control spectacles), Phase 2 (6–12 months; A.R.R.E.S.T. spectacle films), and Phase 3 (12–14 months; bilateral control spectacles) are delineated by shading. Sample sizes (participants) at each time-point are indicated.

Twenty-three children were enrolled, 22 analysed (15 females; 10.7 ± 2.2 years of age; baseline axial length: 23.59 ± 0.61 mm; Table 1). No adverse events occurred; HCVA at dispensing was within 1 line of best-corrected acuity. Six-month axial elongation was significantly lower in Phase 2 than Phase 1: estimated marginal mean 0.057 mm (95% CI 0.013 to 0.102) versus 0.102 mm (0.058 to 0.146); difference −0.045 mm ( − 0.066 to −0.024), *p* < 0.001. Growth slopes declined from 0.017 to 0.011 mm/month (Δ of −0.006 mm/month, *p* < 0.001). Group-level axial-length trajectories are shown in Fig. 1. Matched 2-month increments were 0.027 mm (Phase 1), 0.015 mm (Phase 2), and 0.026 mm (Phase 3). Post-cessation growth did not exceed baseline (Phase 3 versus Phase 1: −0.001 mm, *p* = 0.37), confirming no rebound.

**Table 1:** Baseline characteristics, including subjective refraction (OD/OS), axial length, age at baseline, ethnicity, parental myopia and average lens wear time in phases 1 and 2 for all participants.

Participant ID	BL Subjective Refraction OD (Sph/Cyl × Axis)	BL Subjective Refraction OS (Sph/Cyl × Axis)	BL Axial Length (mm) OD/OS	Age at BL (y)/Sex	Ethnicity	Parental Myopia	Phase 1 Wear (hrs/wk)	Phase 2 Wear (hrs/wk)
P01	–v1.00/–0.25 × 065	–1.25/–0.50 × 025	22.540/22.766	8.8/F	Other	Neither	87.9	80.9
P02	–2.50 DS	–2.50 DS	23.750/23.700	14.8/F	Caucasian	Mother	105.4	74.6
P03	–1.25 DS	–1.50 DS	24.008/24.240	10.9/F	Caucasian	Mother	95.7	81.6
P04 (D/C)	–1.00/–0.25 × 110	–1.00 DS	22.806/22.710	11.5/F	Caucasian	Mother	67.6	50.8
P05	–1.00 DS	–0.75 DS	24.522/24.528	9.2/M	Caucasian	Father	60.6	50.7
P06	–3.25/–1.00 × 020	–1.25/–1.00 × 170	23.670/23.188	9.6/F	Caucasian	Both	80.2	68.0
P07	–3.50/–0.75 × 002	–2.50/–0.50 × 167	24.200/23.720	8.8/M	Other	Mother	91.1	100.0
P08	–2.75/–0.50 × 090	–3.00/–0.75 × 092	25.100/25.064	15.4/M	Caucasian	Mother	84.0	88.6
P10	–1.50 DS	–2.50/–0.25 × 115	23.320/23.912	11.1/F	Caucasian	Father	89.0	84.0
P11 (D/C)	–0.50/–0.25 × 150	–0.75/–0.25 × 040	23.210/23.296	7.7/F	Caucasian	Both	43.3	56.0
P12	–2.00 DS	–1.50/–0.50 × 050	23.412/23.168	13.3/F	Asian	Neither	30.0	30.0
P13	–1.25 DS	–0.75/–0.50 × 088	23.060/23.058	11.8/F	Caucasian	Mother	93.6	101.2
P14	–0.50 DS	–1.50 DS	23.516/23.964	10.5/M	Asian	Mother	32.4	49.9
P15	–1.50/–0.25 × 068	–1.50/–0.25 × 092	23.604/23.516	10.6/F	Other	Both	83.1	99.0
P16	–0.50/–0.25 × 165	–0.75/–0.50 × 005	22.816/22.780	11.5/M	Asian	Mother	68.2	98.8
P17	–0.50 DS	–0.50 DS	23.822/23.644	7.0/M	Asian	Mother	27.0	30.9
P18	–0.50/–0.25 × 090	–0.75 DS	23.426/23.542	13.2/F	Caucasian	Neither	73.7	105.0
P19	–1.75 DS	–1.75 DS	22.790/22.820	7.7/F	Asian	Neither	98.0	98.0
P20	–1.25/–0.50 × 083	–0.75/–0.50 × 098	23.322/23.298	10.4/F	Caucasian	Mother	71.8	105.0
P21	–1.25 DS	–0.50 DS	24.180/24.026	12.2/F	Asian	Mother	98.0	96.8
P22	–2.50/–0.50 × 090	–2.25/–0.50 × 090	24.236/24.184	9.9/M	Asian	Mother	77.0	89.8
P23	–2.75/–0.75 × 090	–2.00/–0.75 × 100	24.164/23.912	10.4/F	Caucasian	Neither	88.8	100.4

*BL* baseline, *OD* right eye, *OS* left eye, *Sph* sphere, *Cyl* cylinder, *DS* dioptre sphere, *D/C* discontinued (participant withdrew before study completion); Phase 1 = 0 to 6 months (single–vision control spectacles); Phase 2 = 6 to 12 months (A.R.R.E.S.T. spectacles). P09 was excluded from the analysis, as the optical biometer at the study site was changed during the trial and differences between instruments may have affected axial length measurement comparison.

## Discussion and conclusion

Axial elongation was lower in the test phase; post-cessation growth returned to baseline without rebound. As control always preceded test, the −0.045 mm difference carries expected contributions from age-related deceleration and seasonal variation. The absence of a concurrent randomised control group limits causal inference. Refractive measurements were non-cycloplegic; cycloplegic confirmation is warranted in future trials. This approach differs from defocus- or scatter-based lenses by preserving single-vision correction and targeting temporal contrast modulation.

Together with the published contact-lens pilot study [5], these data provide a dual-modality safety foundation for single vision lenses featuring opaque non-refractive features. Wear-time adherence was heterogeneous (Table 1), precluding dose-response evaluation. The directional efficacy signals warrant deeper investigation in a properly powered randomised controlled trial, now underway.

Recent reports show that some defocus-based spectacle designs produce retinal shadows [6], raising the hypothesis that spatiotemporal contrast modulation may contribute to therapeutic effects via mechanisms analogous to the proposed temporal contrast signal; testing this requires dedicated retinal imaging and electrophysiological studies.

## Supplementary information

Supplementary Figure 1

Supplementary Figure 1 legend

## Data Availability

De-identified individual participant records for axial length and spectacle lens adherence, as well as the study protocol, will be made available upon reasonable request to the corresponding author. Access is subject to execution of a data confidentiality agreement with nthalmic Pty Ltd.
