# Supplementary material for: Spectacles with non‑refractive opaque features for paediatric myopia: a safety pilot trial
Source: Eye (Lond). 2026 Aug 14;40(15):2476–9. doi: 10.1038/s41433-026-04795-5 (PMC13675195; doi:10.1038/s41433-026-04795-5)
Supplement: Supplementary file 1 [file 41433_2026_4795_MOESM2_ESM.docx]

***Supplementary Figure 1****: Flowchart showing the disposition of participants throughout the clinical trial, including the reasons for discontinuation.*
